# Cost of organ/space infection in elective colorectal surgery. Is it just a problem of rates?

**DOI:** 10.1186/2047-2994-4-S1-P77

**Published:** 2015-06-16

**Authors:** E Shaw, A Gomila, M Piriz, F Obradors, R Escofet, R Vazquez, JM Badia, L Martin, D Fraccalvieri, M Brugués, MC Nicolás, E Espejo, A Castro, A Cruz, E Limón, F Gudiol, M Pujol

**Affiliations:** 1 Hospital Universitari de Bellvitge, L’Hospitalet de Llobregat, Barcelona, Spain; 2 Hospital Parc Taulí, Sabadell, Barcelona, Spain; 3 Fundació Althaia de Manresa, Manresa, Barcelona, Spain; 4 Fundació Hospital Asil de Granollers, Granollers, Barcelona, Spain; 5 Hospital de Viladecans, Viladecans, Barcelona, Spain; 6 Consorci Sanitari de l'Anoia, Igualada, Barcelona, Spain; 7 Hospital Universitari Mutua de Terrassa, Terrassa, Barcelona, Spain; 8 Consorci Sanitari de Terrassa, Terrassa, Barcelona, Spain; 9 Hospital Universitari Sant Joan de Reus, Reus, Tarragona, Spain; 10 Hospital General-Parc Sanitari Sant Joan de Déu, Saint Boi de Llobregat, Barcelona, Spain; 11 VINCat Program, Departament de Salut, Hospitalet de Llobregat, Barcelona, Spain

## Introduction

Organ/space (O/S) infection in colorectal surgery remains a major health problem. In Catalonia, the VINCat Program has monitored 24,832 procedures during 2007-2014, showing a steady rate of O/S infection over the years, 8.2% (95% CI 7.9 - 8.6). Improving awareness of stakeholders could be an easy strategy for assembling quality programs within health systems.

## Objectives

Evaluating excess costs of organ/space infections associated with elective colorectal surgery in the Catalan Health System.

## Methods

We selected a sample of 10 different sized hospitals that provided data to VINCat from January 2012-June 2014. To estimate the excess of cost we based on differences between lengths of stay in patients with and without O/S infection and extra-cost related to readmission/need of ICU and re-operation.

## Results

A total of 2276 patients underwent elective colorectal surgery. O/S infection occurred in 193 (8.5%). Patients with O/S were more frequently men (73% versus 60%; p = 0.001); underwent a rectal procedure (43% versus 31%; p<0.001) and had NNIS index ≥ 1 (43% versus 34%; p = 0.06). Median length of stay was 3 fold higher (22 days versus 7 days; p < 0.001) when O/S occurred which accounted for an extra cost of €3,052 per patient. Within the group of O/S infection, 45/193(23%) patients were re-admitted with a median length of stay of 13 days [IQR 8 - 17)]; 117/193 (60%) required re-operation and 56/193 (29%) required intensive care unit stay with a median length of stay of 5 days [IQR 3 - 12]. This added an additional cost of €2,235 per patient. Accordingly, 193 O/S infections accounted for an overall excess cost of €1,020,391.

## Conclusion

O/S infection represents an important excess of cost for the Catalan Health System. The reinforcement of quality strategies can lead to a strong reduction in the use of resources and relieve its current financial constraints.

## Disclosure of interest

None declared.

